# Dataset for transcriptomic profiles associated with development of sexual structures in *Aspergillus flavus*

**DOI:** 10.1016/j.dib.2022.108033

**Published:** 2022-03-14

**Authors:** Jane Marian Luis, Ignazio Carbone, Brian M. Mack, Matthew D. Lebar, Jeffrey W. Cary, Matthew K. Gilbert, Deepak Bhatnagar, Carol-Carter Wientjes, Gary A. Payne, Geromy G. Moore, Yaken Obaydeh Ameen, Peter S. Ojiambo

**Affiliations:** a Center for Integrated Fungal Research, Department of Entomology and Plant Pathology, North Carolina State University, Raleigh, NC 27695, United States; b United States Department of Agriculture, Agricultural Research Service, Southern Regional Research Center, New Orleans, LA 70124, United States

**Keywords:** Fertility, Sclerotia, Sexual development, Transcriptional analysis

## Abstract

Information on the transcriptomic changes that occur within sclerotia of *Aspergillus flavus* during its sexual cycle is very limited and warrants further research. The findings will broaden our knowledge of the biology of *A. flavus* and can provide valuable insights in the development or deployment of non-toxigenic strains as biocontrol agents against aflatoxigenic strains. This article presents transcriptomic datasets included in our research article entitled, “Development of sexual structures influences metabolomic and transcriptomic profiles in *Aspergillus flavus*” [1], which utilized transcriptomics to identify possible genes and gene clusters associated with sexual reproduction and fertilization in *A. flavus*. RNA was extracted from sclerotia of a high fertility cross (Hi-Fert-Mated), a low fertility cross (Lo-Fert-Mated), and unmated strains (Hi-Fert-Unmated and Lo-Fert-Unmated) of *A. flavus* collected immediately after crossing and at every two weeks until eight weeks of incubation on mixed cereal agar at 30 °C in continuous darkness (*n* = 4 replicates from each treatment for each time point; 80 total). Raw sequencing reads obtained on an Illumina NovaSeq 6000 were deposited in NCBI's Sequence Read Archive (SRA) repository under BioProject accession number PRJNA789260. Reads were mapped to the *A. flavus* NRRL 3357 genome (assembly JCVI-afl1-v2.0; GCA_000006275.2) using STAR software. Differential gene expression analyses, functional analyses, and weighted gene co-expression network analysis were performed using DESeq2 R packages. The raw and analyzed data presented in this article could be reused for comparisons with other datasets to obtain transcriptional differences among strains of *A. flavus* or closely related species. The data can also be used for further investigation of the molecular basis of different processes involved in sexual reproduction and sclerotia fertility in *A. flavus*.

**Specifications Table**

**Table:** 

Subject	Biological Sciences (Omics: Transcriptomics)
Specific subject area	Mycology
Type of data	Table
How the data were acquired	Sclerotium samples were sent to the North Carolina State University Genomic Sciences Laboratory (Raleigh, NC, USA) for RNA extraction using a Qiagen RNeasy mini kit followed by Illumina RNA library construction and sequencing on an Illumina NovaSeq 6000
Data format	Raw, Analyzed, Filtered
Description of data collection	RNA was isolated from sclerotia of *A. flavus* of a high fertility cross (Hi-Fert-Mated), a low fertility cross (Lo-Fert-Mated) and unmated strains (Hi-Fert-Unmated and Lo-Fert-Unmated) at five consecutive time-points starting from immediately after crossing until eight weeks of incubation
Data source location	Institution: United States Department of Agriculture, Agricultural Research Service, Southern Regional Research Center City/Town/Region: New Orleans, LA Country: United States
Data accessibility	Repository name for sequence reads: NCBI Sequence Read Archive (SRA) Data identification number: PRJNA789260 Direct URL to data: https://www.ncbi.nlm.nih.gov/sra/?term=PRJNA789260 Repository name for dataset: Mendeley Data Data identification number: 10.17632/2f5s7vv7gn.1 Direct URL to data: https://data.mendeley.com/datasets/2f5s7vv7gn/draft?a=88b5c55f-dc95-49f5-8b72-fe9d94db30d6
Related research article	J.M. Luis, I. Carbone, B.M. Mack, M.D. Lebar, J.W. Cary, M.K. Gilbert, D. Bhatnagar, C.C. Wientjes, G.A. Payne, G.G. Moore, Y.O. Ameen, P.S. Ojiambo, Development of sexual structures influences metabolomic and transcriptomic profiles in *Aspergillus flavus*, Fungal Biology, https://doi.org/10.1016/j.funbio.2022.01.001

**Value of the Data**

• This article reports transcriptomic dataset from sclerotia of *A. flavus* exhibiting high level of fertility and compared to sclerotia with low level of fertility and unfertilized sclerotia. The data will be useful for researchers interested in the gene expression, genomics and functional genomics of *A. flavus* and other fungi with a sexual cycle.
• The raw data and methodologies in this article could be reused to compare with other similar datasets to obtain transcriptional differences among strains of *A. flavus* or closely related species.
• The reported data can be used to screen for candidate genes that are involved in the initiation of sexual reproduction, development of sexual structures, and other fertilization-associated processes in *A. flavus*. It can be further used in investigating the molecular basis and functional pathways of these processes.
• Genes that are differentially expressed between treatments and time points can be used as markers for sclerotia fertility and can be useful in developing biocontrol strategies against aflatoxigenic strains of *A. flavus*.

## 1 Data Description

A total of 80 transcriptome libraries were generated from four samples collected from each of four treatments (Hi-Fert-Mated, Lo-Fert-Mated, Hi-Fert-Unmated and Lo-Fert-Unmated) at five sampling time points (T0, T1, T2, T3 and T4 described below). The sequence reads obtained on an Illumina NovaSeq 6000 were deposited in NCBI's Sequence Read Archive (SRA) repository under BioProject accession number PRJNA789260. A list of samples according to treatment × time point combination is shown in Table 1. The reported values for % duplicates, % GC content, and total sequence lengths have been filtered to remove low-quality reads and adapters from raw sequence data. Each library contains an average of 18.69 million filtered quality reads, yielding a total of 1.50 billion reads (Table 1).

**Table 1:** List of sclerotia samples according to treatment and time point combinations.

Sample Name	Samples (4 replicates per treatment per time point)	% Duplicates	% GC Content	Total Sequences (M)
Hi-Fert-Mated_T0_a	High Fertility Cross (immediately after crossing)	78.30%	50%	19.40
Hi-Fert-Mated_T0_b	High Fertility Cross (immediately after crossing)	76.30%	49%	17.90
Hi-Fert-Mated_T0_c	High Fertility Cross (immediately after crossing)	78.50%	51%	18.10
Hi-Fert-Mated_T0_d	High Fertility Cross (immediately after crossing)	72.70%	50%	14.90
Hi-Fert-Mated_T1_a	High Fertility Cross (2 weeks of incubation)	71.00%	50%	17.50
Hi-Fert-Mated_T1_b	High Fertility Cross (2 weeks of incubation)	72.60%	50%	17.30
Hi-Fert-Mated_T1_c	High Fertility Cross (2 weeks of incubation)	79.70%	50%	10.90
Hi-Fert-Mated_T1_d	High Fertility Cross (2 weeks of incubation)	72.50%	50%	15.80
Hi-Fert-Mated_T2_a	High Fertility Cross (4 weeks of incubation)	75.00%	50%	23.40
Hi-Fert-Mated_T2_b	High Fertility Cross (4 weeks of incubation)	73.50%	50%	19.10
Hi-Fert-Mated_T2_c	High Fertility Cross (4 weeks of incubation)	84.60%	50%	16.40
Hi-Fert-Mated_T2_d	High Fertility Cross (4 weeks of incubation)	76.60%	51%	21.10
Hi-Fert-Mated_T3_a	High Fertility Cross (6 weeks of incubation)	76.50%	51%	18.00
Hi-Fert-Mated_T3_b	High Fertility Cross (6 weeks of incubation)	74.30%	51%	21.50
Hi-Fert-Mated_T3_c	High Fertility Cross (6 weeks of incubation)	79.10%	51%	19.30
Hi-Fert-Mated_T3_d	High Fertility Cross (6 weeks of incubation)	77.00%	51%	17.50
Hi-Fert-Mated_T4_a	High Fertility Cross (8 weeks of incubation)	76.40%	51%	20.40
Hi-Fert-Mated_T4_b	High Fertility Cross (8 weeks of incubation)	75.90%	51%	20.80
Hi-Fert-Mated_T4_c	High Fertility Cross (8 weeks of incubation)	83.50%	51%	16.10
Hi-Fert-Mated_T4_d	High Fertility Cross (8 weeks of incubation)	77.50%	51%	19.30
Lo-Fert-Mated_T0_a	Low Fertility Cross (immediately after crossing)	76.80%	50%	23.90
Lo-Fert-Mated_T0_b	Low Fertility Cross (immediately after crossing)	74.90%	50%	21.50
Lo-Fert-Mated_T0_c	Low Fertility Cross (immediately after crossing)	72.00%	50%	18.00
Lo-Fert-Mated_T0_d	Low Fertility Cross (immediately after crossing)	78.90%	50%	13.70
Lo-Fert-Mated_T1_a	Low Fertility Cross (2 weeks of incubation)	74.70%	51%	19.90
Lo-Fert-Mated_T1_b	Low Fertility Cross (2 weeks of incubation)	73.30%	50%	18.60
Lo-Fert-Mated_T1_c	Low Fertility Cross (2 weeks of incubation)	75.70%	50%	24.30
Lo-Fert-Mated_T1_d	Low Fertility Cross (2 weeks of incubation)	76.10%	50%	20.40
Lo-Fert-Mated_T2_a	Low Fertility Cross (4 weeks of incubation)	73.30%	50%	18.00
Lo-Fert-Mated_T2_b	Low Fertility Cross (4 weeks of incubation)	72.80%	50%	22.70
Lo-Fert-Mated_T2_c	Low Fertility Cross (4 weeks of incubation)	76.70%	50%	16.90
Lo-Fert-Mated_T2_d	Low Fertility Cross (4 weeks of incubation)	80.20%	50%	17.30
Lo-Fert-Mated_T3_a	Low Fertility Cross (6 weeks of incubation)	74.00%	50%	18.30
Lo-Fert-Mated_T3_b	Low Fertility Cross (6 weeks of incubation)	78.20%	51%	21.60
Lo-Fert-Mated_T3_c	Low Fertility Cross (6 weeks of incubation)	77.20%	50%	18.40
Lo-Fert-Mated_T3_d	Low Fertility Cross (6 weeks of incubation)	80.80%	50%	19.60
Lo-Fert-Mated_T4_a	Low Fertility Cross (8 weeks of incubation)	73.40%	51%	18.90
Lo-Fert-Mated_T4_b	Low Fertility Cross (8 weeks of incubation)	75.90%	51%	23.20
Lo-Fert-Mated_T4_c	Low Fertility Cross (8 weeks of incubation)	74.60%	50%	20.50
Lo-Fert-Mated_T4_d	Low Fertility Cross (8 weeks of incubation)	82.80%	50%	20.80
Hi-Fert-Unmated_T0_a	Unmated NRRL 29507 (immediately after crossing)	78.00%	51%	25.30
Hi-Fert-Unmated_T0_b	Unmated NRRL 29507 (immediately after crossing)	78.30%	51%	27.90
Hi-Fert-Unmated_T0_c	Unmated NRRL 29507 (immediately after crossing)	74.10%	50%	22.70
Hi-Fert-Unmated_T0_d	Unmated NRRL 29507 (immediately after crossing)	78.20%	51%	25.50
Hi-Fert-Unmated_T1_a	Unmated NRRL 29507 (2 weeks of incubation)	76.50%	50%	19.20
Hi-Fert-Unmated_T1_b	Unmated NRRL 29507 (2 weeks of incubation)	76.20%	50%	20.10
Hi-Fert-Unmated_T1_c	Unmated NRRL 29507 (2 weeks of incubation)	76.20%	50%	19.30
Hi-Fert-Unmated_T1_d	Unmated NRRL 29507 (2 weeks of incubation)	78.00%	50%	17.30
Hi-Fert-Unmated_T2_a	Unmated NRRL 29507 (4 weeks of incubation)	88.20%	51%	19.60
Hi-Fert-Unmated_T2_b	Unmated NRRL 29507 (4 weeks of incubation)	76.60%	51%	18.40
Hi-Fert-Unmated_T2_c	Unmated NRRL 29507 (4 weeks of incubation)	82.70%	50%	18.00
Hi-Fert-Unmated_T2_d	Unmated NRRL 29507 (4 weeks of incubation)	78.00%	51%	18.00
Hi-Fert-Unmated_T3_a	Unmated NRRL 29507 (6 weeks of incubation)	77.50%	51%	17.00
Hi-Fert-Unmated_T3_b	Unmated NRRL 29507 (6 weeks of incubation)	77.60%	51%	19.10
Hi-Fert-Unmated_T3_c	Unmated NRRL 29507 (6 weeks of incubation)	80.10%	51%	16.80
Hi-Fert-Unmated_T3_d	Unmated NRRL 29507 (6 weeks of incubation)	81.50%	51%	17.40
Hi-Fert-Unmated_T4_a	Unmated NRRL 29507 (8 weeks of incubation)	88.20%	51%	18.60
Hi-Fert-Unmated_T4_b	Unmated NRRL 29507 (8 weeks of incubation)	80.90%	51%	17.50
Hi-Fert-Unmated_T4_c	Unmated NRRL 29507 (8 weeks of incubation)	81.30%	51%	17.60
Hi-Fert-Unmated_T4_d	Unmated NRRL 29507 (8 weeks of incubation)	85.30%	51%	19.10
Lo-Fert-Unmated_T0_a	Unmated NRRL 21882 (immediately after crossing)	76.60%	50%	16.80
Lo-Fert-Unmated_T0_b	Unmated NRRL 21882 (immediately after crossing)	77.00%	50%	17.20
Lo-Fert-Unmated_T0_c	Unmated NRRL 21882 (immediately after crossing)	77.70%	50%	18.20
Lo-Fert-Unmated_T0_d	Unmated NRRL 21882 (immediately after crossing)	73.40%	50%	13.80
Lo-Fert-Unmated_T1_a	Unmated NRRL 21882 (2 weeks of incubation)	75.60%	50%	13.60
Lo-Fert-Unmated_T1_b	Unmated NRRL 21882 (2 weeks of incubation)	77.50%	50%	17.60
Lo-Fert-Unmated_T1_c	Unmated NRRL 21882 (2 weeks of incubation)	75.90%	49%	15.00
Lo-Fert-Unmated_T1_d	Unmated NRRL 21882 (2 weeks of incubation)	78.30%	49%	12.80
Lo-Fert-Unmated_T2_a	Unmated NRRL 21882 (4 weeks of incubation)	76.70%	50%	13.00
Lo-Fert-Unmated_T2_b	Unmated NRRL 21882 (4 weeks of incubation)	72.30%	51%	14.60
Lo-Fert-Unmated_T2_c	Unmated NRRL 21882 (4 weeks of incubation)	83.20%	50%	19.20
Lo-Fert-Unmated_T2_d	Unmated NRRL 21882 (4 weeks of incubation)	82.20%	50%	16.90
Lo-Fert-Unmated_T3_a	Unmated NRRL 21882 (6 weeks of incubation)	78.00%	51%	21.50
Lo-Fert-Unmated_T3_b	Unmated NRRL 21882 (6 weeks of incubation)	83.70%	51%	22.60
Lo-Fert-Unmated_T3_c	Unmated NRRL 21882 (6 weeks of incubation)	74.60%	50%	18.70
Lo-Fert-Unmated_T3_d	Unmated NRRL 21882 (6 weeks of incubation)	71.50%	50%	18.40
Lo-Fert-Unmated_T4_a	Unmated NRRL 21882 (8 weeks of incubation)	69.90%	50%	18.90
Lo-Fert-Unmated_T4_b	Unmated NRRL 21882 (8 weeks of incubation)	72.80%	50%	16.70
Lo-Fert-Unmated_T4_c	Unmated NRRL 21882 (8 weeks of incubation)	75.40%	50%	16.90
Lo-Fert-Unmated_T4_d	Unmated NRRL 21882 (8 weeks of incubation)	71.90%	51%	17.30

**Sum:**				1495.30

**Mean:**				18.69

Multifactor analyses were used to identify genes that were differentially expressed between main factor effects: fertility (high vs. low), sampling time points vs. T0 (2 weeks incubation vs. T0, until 8 weeks incubation vs. T0), and mating (mated = TRUE vs. unmated = FALSE). Analyses were conducted in DeSeq2 using the formula: ∼ time + fertility + mating. Differentially expressed genes were defined as having a fold change of 2 and an adjusted *p*-value < 0.05. Expression values for genes that meet these criteria are listed in Table 2. A total of 2804 DEGs were identified between fertility levels, up to 3810 DEGs between sampling time points, and 731 DEGs between mating categories (Table 2A and 2B). The interaction effect between fertility and mating was investigated using the formula:  ∼ time + fertility + mating + fertility:mating and can be identified in the dataset as Fertilityhigh.matedTRUE. This analysis identified 710 DEGs that were detected in Hi-Fert-Mated but not in Lo-Fert-Mated (Table 2A and 2B). All DEGs identified in the multifactor comparisons were subjected to functional enrichment analysis. *P*-values for each functional term were reported, with separate tables for both up-regulated and down-regulated genes (Table 2C), up-regulated genes only (Table 2D), and down-regulated genes only (Table 2E). Pairwise analyses between 36 different treatment × time point combinations were also evaluated. These comparisons identified genes that were differentially expressed between mated strains at similar time points, unmated strains at similar time points, and similar treatments at consecutive time points. Number of DEGs for the pairwise comparisons ranged from 2 to 3058 genes (Table 2F and 2G).

**Table 2:** Number of differentally expressed genes obtained from pairwise analyses of different treatment andtime point combinations.

Comparison	Upregulated	Downregulated	Total
Hi-Fert-Mated T0 vs Lo-Fert-Mated T0	914	2048	2962
Hi-Fert-Mated T1 vs Lo-Fert-Mated T1	153	695	848
Hi-Fert-Mated T2 vs Lo-Fert-Mated T2	427	1504	1931
Hi-Fert-Mated T3 vs Lo-Fert-Mated T3	1124	1909	3033
Hi-Fert-Mated T4 vs Lo-Fert-Mated T4	595	1754	2349
Hi-Fert-Unmated T0 vs Lo-Fert-Unmated T0	810	2248	3058
Hi-Fert-Unmated T1 vs Lo-Fert-Unmated T1	673	1705	2378
Hi-Fert-Unmated T2 vs Lo-Fert-Unmated T2	970	2046	3016
Hi-Fert-Unmated T3 vs Lo-Fert-Unmated T3	703	1891	2594
Hi-Fert-Unmated T4 vs Lo-Fert-Unmated T4	643	1904	2547
Hi-Fert-Mated T1 vs Hi-Fert-Mated T0	132	1336	1468
Hi-Fert-Mated T2 vs Hi-Fert-Mated T1	204	810	1014
Hi-Fert-Mated T3 vs Hi-Fert-Mated T2	274	254	528
Hi-Fert-Mated T4 vs Hi-Fert-Mated T3	9	234	243
Lo-Fert-Mated T1 vs Lo-Fert-Mated T0	481	1620	2101
Lo-Fert-Mated T2 vs Lo-Fert-Mated T1	55	407	462
Lo-Fert-Mated T3 vs Lo-Fert-Mated T2	61	270	331
Lo-Fert-Mated T4 vs Lo-Fert-Mated T3	23	93	116
Hi-Fert-Unmated T1 vs Hi-Fert-Unmated T0	229	1149	1378
Hi-Fert-Unmated T2 vs Hi-Fert-Unmated T1	85	509	594
Hi-Fert-Unmated T3 vs Hi-Fert-Unmated T2	27	592	619
Hi-Fert-Unmated T4 vs Hi-Fert-Unmated T3	81	240	321
Lo-Fert-Unmated T1 vs Lo-Fert-Unmated T0	168	1003	1171
Lo-Fert-Unmated T2 vs Lo-Fert-Unmated T1	91	1122	1213
Lo-Fert-Unmated T3 vs Lo-Fert-Unmated T2	14	252	266
Lo-Fert-Unmated T4 vs Lo-Fert-Unmated T3	2	9	11
Hi-Fert-Mated T0 vs Hi-Fert-Unmated T0	43	165	208
Hi-Fert-Mated T1 vs Hi-Fert-Unmated T1	26	117	143
Hi-Fert-Mated T2 vs Hi-Fert-Unmated T2	239	412	651
Hi-Fert-Mated T3 vs Hi-Fert-Unmated T3	771	711	1482
Hi-Fert-Mated T4 vs Hi-Fert-Unmated T4	846	891	1737
Lo-Fert-Mated T0 vs Lo-Fert-Unmated T0	0	2	2
Lo-Fert-Mated T1 vs Lo-Fert-Unmated T1	311	472	783
Lo-Fert-Mated T2 vs Lo-Fert-Unmated T2	233	215	448
Lo-Fert-Mated T3 vs Lo-Fert-Unmated T3	130	28	158
Lo-Fert-Mated T4 vs Lo-Fert-Unmated T4	451	441	892

Co-expression module analysis using weighted gene co-expression network analysis (WGCNA) identified 25 modules of highly correlated genes in the Hi-Fert strain (NRRL 29507) (Table 3A). By default, WGCNA uses colors to name the modules. The overall gene expression profile of each module was correlated with mating and time point. Black, dark red, salmon, and pink modules yielded correlation values with mating above 0.5 (*p*-value < 0.05) (Table 3A). The preservation (*Z*) score shows how strong the modules in the Hi-Fert strain are preserved among genes in the Lo-Fert strain (NRRL 21882). Values between 2 and 10 were considered as weak to moderately preserved modules, while *Z* scores above 10 were considered as highly preserved. The pink module was highly preserved while the other three modules in the low fertility strains were low to moderately preserved (Table 3A). Results of the enrichment analysis for these four modules are shown in Table 3B. The list of genes for each co-expression module is shown in Table 3C.

## 2 Experimental Design, Materials and Methods

### 2.1 Treatments and sclerotia production

This article reports the transcriptomes of *A. flavus* sclerotia exhibiting different levels of fertility collected over an eight-week period of incubation at 30 °C in continuous darkness. The treatments consisted of a high fertility cross (Hi-Fert-Mated, NRRL 29507 sclerotia x NRRL 21882 conidia), a low fertility cross (Lo-Fert-Mated, NRRL 21882 sclerotia x NRRL 29507 conidia), and unmated strains (Hi-Fert-Unmated, NRRL 29507 sclerotia; Lo-Fert-Unmated, NRRL 21882 sclerotia) of *A. flavus*. Selection of parental strains (NRRL 29507 and NRRL 21882) was based on the study of Horn et al. [2], and the sclerotia and conidia from parental strains were prepared according to the methodologies in Luis et al. [1,3]. Briefly, Hi-Fert-Mated was prepared by placing sclerotia of NRRL 29507 over a layer of NRRL 21882 conidia on mixed cereal agar (MCA) [4] plate. Lo-Fert-Mated was prepared by placing sclerotia of NRRL 21882 over a layer of NRRL 21882 conidia on MCA plate. Sclerotia of NRRL 29507 and of NRRL 21882 were individually plated on MCA to serve as unmated controls. Culture plates were sealed with parafilm, arranged in zip lock bags, and then incubated at 30 °C under continuous darkness.

Changes in transcription profiles over the eight-week period were assessed by collecting culture plates from each treatment starting from immediately after crossing (T0), and at 2 weeks (T1), 4 weeks (T2), 6 weeks (T3) and 8 weeks (T4) of incubation. During harvesting, 3–5 mL distilled water containing 0.01% Triton-X was poured onto the culture plate and then the sclerotia were carefully detached from the agar using a transfer loop. Residual conidia that remained attached to the sclerotia were removed by transferring the sclerotia to 50 mL microtubes, repeatedly washed in DEPC-treated water, then filtered through a miracloth (MilliporeSigma). The sclerotium samples (*n* = 4 sample replicates per treatment per time point; 80 total) were flash frozen, stored in -80 °C until all samples were collected, then submitted to the North Carolina State University Genomic Sciences Laboratory (Raleigh, NC, USA) for RNA extraction and sequencing.

### 2.2 RNA extraction, library construction and Illumina sequencing

RNA extraction was performed using Qiagen RNeasy mini columns and reagents (Germantown, MD, USA) following the manufacturer's instructions. Integrity, purity, and concentration of RNA were checked using an Agilent 2100 Bioanalyzer with an RNA 6000 Nano Chip (Agilent Technologies, USA). Messenger RNA (mRNA) was purified using oligo-dT beads included in the NEBNext Poly(A) mRNA Magnetic Isolation Module (New England Biolabs, USA). Complementary DNA (cDNA) libraries for Illumina sequencing were prepared with the NEBNext Ultra Directional RNA Library Prep Kit (NEB) and NEBNext Multiplex Oligos for Illumina (NEB) following the manufacturer-specific protocol. Amplified library fragments were purified, quality-checked and quantified for final concentration using an Agilent 2200 Tapestation (Agilent Technologies, USA). Quantified libraries were pooled in equimolar amounts for clustering and sequencing on an Illumina NovaSeq 6000 DNA sequencer in 3 XP split lanes using a 100 bp single end sequencing SP reagent kit (Illumina, USA). Raw bcl files were generated via the Real Time Analysis software package, then de-multiplexed by sample into fastq files.

### 2.3 Differential expression analysis

The quality of raw sequence reads was checked using FastQC [5] prior to analysis. Low quality sequences and adapters were removed using BBDuk [6]. The sequencing reads were then mapped to the *A. flavus* NRRL 3357 genome (assembly JCVI-afl1-v2.0; GCA_000006275.2) using STAR v2.6.1 [7]. Multifactor analyses comparing the effects of fertility, mating and sampling time point were conducted in DESeq2 v1.28.1 [8]. Multifactor analyses were modeled using the formula: ∼time + fertility + mating. Interaction effect between fertility and mating was analyzed using the formula: ∼ time + fertility + mating + fertility:mating. Values for the interaction effect were extracted with the “results” function from DESeq2 as “results(dds, name=‘fertilityhigh.matedTRUE’, alpha=0.05)”. Genes with log2(fold_change) ≥ |1| and adjusted *P* ≤ 0.05 were considered as differentially expressed. Pairwise analyses of differentially expressed genes (DEGs) between treatments or time points were also conducted in DESeq2. All differentially expressed gene sets from each multifactor and pairwise comparisons were subjected to functional enrichment analyses using annotation terms from the Gene Ontology, KEGG pathways, SMURF secondary metabolite clusters, Apoplast-p, Signal-p, Effector-p, Deeploc, and Interpro domains.

### 2.4 Weighted gene co-expression network analysis

Variance-stabilized mRNA counts from DESeq2 were used as input for WGCNA [9] to create individual co-expression networks for Hi-Fert (NRRL 29507) and Lo-Fert (NRRL 21882) strains. The two networks were created using 40 samples with NRRL 29507 and 40 samples with NRRL 21882 as sclerotial parents. The settings used for network adjacency matrix creation was “corFnc=‘bicor’, type=‘signed hybrid’, power=10”. Module preservation analysis was conducted with the module Preservation function. Comparison between the Lo-Fert modules and the Hi-Fert modules was used as the reference. Functional enrichment analysis was performed on each co-expression module gene set using annotation terms from the Gene Ontology, KEGG pathways, SMURF secondary metabolite clusters, Apoplast-p, Signal-p, Effector-p, Deeploc, and Interpro domains.

## CRediT authorship contribution statement

**Jane Marian Luis:** Conceptualization, Formal analysis, Investigation, Writing – original draft. **Ignazio Carbone:** Conceptualization, Resources, Writing – review & editing. **Brian M. Mack:** Conceptualization, Formal analysis, Investigation, Writing – review & editing. **Matthew D. Lebar:** Conceptualization, Formal analysis, Investigation, Writing – review & editing. **Jeffrey W. Cary:** Conceptualization, Funding acquisition, Writing – review & editing. **Matthew K. Gilbert:** Writing – review & editing. **Deepak Bhatnagar:** Writing – review & editing. **Carol-Carter Wientjes:** Investigation, Writing – review & editing. **Gary A. Payne:** Conceptualization. **Geromy G. Moore:** Investigation, Writing – review & editing. **Yaken Obaydeh Ameen:** Investigation. **Peter S. Ojiambo:** Conceptualization, Supervision, Funding acquisition, Writing – review & editing.

## Declaration of Competing Interest

The authors declare that they have no known competing financial interests or personal relationships that could have appeared to influence the work reported in this paper.

## Data Availability

Dataset for transcriptomic profiles associated with development of sexual structures in Aspergillus flavus (Original data) (Mendeley Data).

## Appendix Supplementary materials

Image, application 1
